# What Did You Google? Describing Online Health Information Search Patterns of ED patients and Their Relationship with Final Diagnoses

**DOI:** 10.5811/westjem.2017.5.34108

**Published:** 2017-07-14

**Authors:** Danielle M. McCarthy, Grant N. Scott, D. Mark Courtney, Alyssa Czerniak, Amer Z. Aldeen, Stephanie Gravenor, Scott M. Dresden

**Affiliations:** * Northwestern University, Feinberg School of Medicine, Department of Emergency Medicine, Chicago, Illinois; † University of New Mexico, Department of Emergency Medicine, Albuquerque, New Mexico; ‡ US Acute Care Solutions, Center for Emergency Medical Education, Canton, Ohio

## Abstract

**Introduction:**

Emergency department (ED) patients’ Internet search terms prior to arrival have not been well characterized. The objective of this analysis was to characterize the Internet search terms patients used prior to ED arrival and their relationship to final diagnoses.

**Methods:**

We collected data via survey; participants listed Internet search terms used. Terms were classified into categories: symptom, specific diagnosis, treatment options, anatomy questions, processes of care/physicians, or “other.” We categorized each discharge diagnosis as either symptom-based or formal diagnosis. The relationship between the search term and final diagnosis was assigned to one of four categories of search/diagnosis combinations (symptom search/symptom diagnosis, symptom search/formal diagnosis, diagnosis search/symptom diagnosis, diagnosis search/formal diagnosis), representing different “trajectories.”

**Results:**

We approached 889 patients; 723 (81.3%) participated. Of these, 177 (24.5%) used the Internet prior to ED presentation; however, seven had incomplete data (N=170). Mean age was 47 years (standard deviation 18.2); 58.6% were female and 65.7% white. We found that 61.7% searched symptoms and 40.6% searched a specific diagnosis. Most patients received discharge diagnoses of equal specificity as their search terms (34% flat trajectory-symptoms and 34% flat trajectory-diagnosis). Ten percent searched for a diagnosis by name but received a symptom-based discharge diagnosis with less specificity. In contrast, 22% searched for a symptom and received a detailed diagnosis. Among those who searched for a diagnosis by name (n=69) only 29% received the diagnosis that they had searched.

**Conclusion:**

The majority of patients used symptoms as the basis of their pre-ED presentation Internet search. When patients did search for specific diagnoses, only a minority searched for the diagnosis they eventually received.

## INTRODUCTION

The Internet has become an important source of health information for patients. According to the most recent Pew Internet and American Life Project national survey (2013), 81% of adults use the Internet and 72% looked up online health information in the preceding year.^1^ Although many of these online health searches may be more general or related to an already-diagnosed condition or planned treatment, 35% of Americans reported looking online specifically to determine what medical condition they may have; 46% of those online diagnosers reported that the information they found online led them to think they needed medical attention.^1^

Within the context of emergency medicine, previous studies asked patients if the health information they found online made them more likely seek care in the emergency department (ED). Among ED patients with Internet access, estimated rates of Internet searches prior to ED presentation varied from 15.1% to 47%.^2^–^4^ Many companies and health systems have produced online “symptom-checking” websites to harness these searches and attempt to improve self-triage, with variable success.^5^ On a population level, healthcare website traffic measurements have been used to forecast ED visit volume.^6^ Similarly, epidemiologic trends for certain conditions such as influenza correlated well with Internet searches for related symptoms.^7^ These prior studies suggest that patient Internet use affects patient concerns, and impacts their choice to seek medical care.

What remains unexplored in the current literature is exactly what individual patients are searching prior to their ED visit. We believe it is important for the emergency physician (EP) to understand what the patient is seeking with an Internet search because an awareness of these patient concerns may inform the conversations and counseling in the ED. What types of information are patients seeking when they turn to the Internet and how does their ultimate diagnosis relate to their original search? We sought to answer these questions through a qualitative analysis.

## METHODS

This analysis is part of a larger prospective survey study focused on how patients use the Internet and their primary physician for health information prior to an ED visit.^8^ This study uses qualitative methods to further analyze the responses of participants who conducted an Internet search prior to visiting the ED (data from primary study reported separately).

### Participants and Procedure

Data collection occurred at an urban academic medical center (>88,000 annual patient visits) with patients enrolled from May 23, 2014, to July 21, 2014. Trained research assistants (RAs) enrolled patients on weekdays 9am–9pm and Saturday 9am–5pm, based on RA availability. All adult patients (age >17) were eligible. The larger study specifically investigated differences in access to health information between adult and geriatric patients. Therefore, there was intentional oversampling of the geriatric population to achieve a balance between geriatric and adult patients; sample-size calculations targeted a total enrollment of 720 participants based on the primary outcome of the larger study. The exclusion criteria included an inability to complete the written survey for any reason (e.g., physical impairment, clinical condition, language barrier).

Population Health Research Capsule

What do we already know about this issue?

The Internet is an important source of health information, and prior studies estimate up to half of ED patients search the Internet prior to ED presentation.

What was the research question?

We sought to describe what types of information patients are searching for in their pre-ED Internet searches.

What was the major finding of the study?

Patients searched for symptoms more often than diagnoses. Correlation between search and ED diagnosis was poor.

How does this improve population health?

Many discharged patients have symptom-based diagnoses (similar to pre-ED symptom-searches). Discussing the lack of a formal diagnosis may be warranted.

Participants provided written informed consent. RAs administered the survey on paper, and it took approximately five minutes to complete. Participants received compensation with a $5 gift card. RAs later entered data into REDCap, a secure, web-based application designed to support data capture for research. The institutional review board approved all study procedures.

### Survey Measures

This qualitative analysis includes the subset of patients from the larger study who answered “yes” when asked, “Before coming to the emergency room today, did you search the Internet about your current symptoms or condition?” As a follow-up question, patients listed the search terms entered and the Internet site(s) visited to look up the information. The top six most frequently accessed health websites were listed, as well as a free-text space for “other” websites.^9^ Additionally, the survey contained questions regarding demographic information (age, sex, race/ethnicity), socioeconomic information (education, household income), access to a primary care physician, and questions related to number of devices owned capable of accessing the Internet. Following survey completion, we extracted additional patient-specific clinical data from the electronic medical record (EMR), including triage acuity, chief complaint, ED disposition (admission, discharge), and ED discharge diagnosis.

### Qualitative Measures and Analysis

Three qualitative metrics are reported. First, based on a review of the literature, we developed an a priori coding schema to categorize the Internet search terms based on content.^10^–^12^ The categories included search terms related to a symptom, a specific diagnosis, treatment options, anatomy, processes of care or physicians, or “other.” Table 2 contains a detailed definition of each category.

The second qualitative analysis phase investigated the relationship between the patients’ initial search term and their final ED diagnosis for those patients who had searched either a symptom or a diagnosis. Many discharge diagnoses in the ED are, in fact, “symptom-based” (e.g., chest pain) as opposed to a more “formal” diagnosis (e.g., myocardial infarction). Therefore, we divided final diagnoses into two large groups: symptom-based diagnoses and formal diagnoses. We considered an ED discharge diagnosis a symptom-based diagnosis if it met one of two criteria: 1) *ICD-9* code range 780–799 (symptoms, signs and ill-defined conditions) (e.g., malaise, abdominal pain, fever, rash); or 2) it named an anatomical body part followed by pain (e.g., ankle pain, wrist pain). Therefore, every encounter received a designation in one of these two categories, either the more general symptom-based diagnosis group or the more specific formal diagnosis group.

After the designation of symptom-based or formal diagnosis, we assigned the relationship between the search term and diagnosis to one of four categories of search/diagnosis combinations representing different “trajectories.” If patients had more than one search term listed, the analysis used the most specific search term. (Diagnosis searches were rated as being more specific than symptom searches.) This analysis excluded patients who had only used search terms related to treatment, anatomy, ED processes, or “other.” A patient’s trajectory from pre-ED presentation Internet search to post-ED-care doctor-assigned discharge diagnosis was defined as *flat trajectory—symptoms* if they searched for a symptom and received a final diagnosis of a symptom. It was defined as *flat trajectory—diagnosis* if they searched for a diagnosis and received a final formal diagnosis. Patients’ diagnosis search term accuracy was recorded. If a patient searched for a symptom and ultimately received a formal diagnosis, they were categorized as having *general to specific trajectory*, whereas if they searched for a specific diagnosis by name and left the ED with a symptom-based diagnosis they were categorized as having a *specific to general trajectory*.

The two qualitative analyses described above examine the bookends of the visit, the initial search and the final diagnosis. An additional analysis conducted describes an intermediate step in the process—the chief complaint. Although it may be of interest to know how the patient’s search influenced the wording of his/her presenting complaint, the concordance between search term and chief complaint *was not* examined because of concerns that chief complaints were potentially influenced by nurse interpretation. The EMR allowed for free-text entry of the chief complaint or for selection from a drop-down menu by the triage nurse; therefore, the chief complaint may not have fully captured the patient’s concern at the time of presentation. For example, “I’m having chest pain, I’m worried it is a heart attack” may have been recorded as “chest pain.” This limitation did not allow for use of the chief complaint as a proxy measure for how the Internet search may have influenced the patient’s statement of his complaint. However, of interest from the *physician perspective*, we assessed the concordance between the chief complaint and the final diagnosis. Even allowing for nursing influence on the chief complaint, the chief complaint recorded in the record was the first introduction that the physician had to the patient, and therefore we assessed the concordance between the complaint and the final diagnosis. Table 4 defines the concordance scale and provides examples.

Two coders analyzed all cases independently. A kappa analysis for a 10% random subsample of cases ensured reliability prior to coding the entire sample. The coders reconciled all disagreements through discussion and selected a final code through consensus. Frequencies are reported for demographic characteristics and for each of the codes. The Fischer’s exact test assessed the association between demographic variables and Internet search terms. All analyses were conducted using STATA software version 13.1 (StataCorp, College Station, TX). We could not pre-determine optimal power or estimate an effect size for this study because the qualitative analysis was exploratory and not testing a quantifiable hypothesis.

## RESULTS

Of the 723 participants, 177 (24%) who completed the larger study searched the Internet prior to ED presentation. Seven participants had incomplete data, resulting in a final sample of 170 (see Figure). The participants had a mean age of 47 years (standard deviation 18.2) and slightly more than half were female (58.6%). The vast majority owned at least one device capable of Internet access (98.2%) (Table 1).

In our sample, 32% (N=55) reported using more than one search term, resulting in a total of 243 search terms. When conducting their Internet searches, the majority of search terms focused on symptoms (54.7%) rather than a diagnosis by name (31.7%) (Table 2). Participants accessed a variety of websites to gather information; 58% of the sample reported searching on WebMD, followed by 40% using the Mayo Clinic website. Although not a formal option on the survey instrument, 37 patients (21.8%) wrote in the “other” category that they simply conducted a Google search and looked at the top hits.

Overall, 56% of the sample left the ED with a formal diagnosis and the remaining 44% received a symptom-based diagnosis upon discharge. The second qualitative analysis excluded 13 patients because they only searched for treatment, anatomy, ED processes or “other,” resulting in a sample of 157 patients. Looking specifically at the relationship between pre-ED presentation Internet search terms and final ED diagnosis, the largest grouping of patients appeared in the *flat trajectory-symptoms* category (34%, 95% confidence interval [CI] for proportions: [27%–41%]). These patients searched for a symptom and were discharged (or admitted) with a diagnosis of a symptom. Approximately a fifth of patients (22%, 95% CI [16%–28%]) had a *general-to-specific* trajectory. For those categorized as *flat trajectory-diagnosis,* 20 patients (13%, 95% CI [8%–18%]) had perfect accuracy in their Internet search (having searched for a diagnosis and received the same, correct final diagnosis). Although this trajectory was flat, it was accurate. In contrast, 33 patients (21%, 95% CI [15%–27%]) searched for a diagnosis and received a different formal diagnosis. Finally, 16 patients (10%, 95% CI [5%–15%]) had a *specific-to-general* trajectory wherein they searched for a specific diagnosis and left the ED with a symptom-based diagnosis (Table 3). Among all of the patients who searched for a diagnosis by name (n=69), 23% received a symptom-based final diagnosis, 48% received a different detailed final diagnosis, and only 29% received the diagnosis that they had searched.

In nearly two-thirds of cases, the chief complaint and final diagnosis showed near or complete concordance (Table 4). This does not, however, imply that a formal diagnosis was made in all of these cases. For example, a chief complaint of “chest pain” and a final diagnosis (symptom-based) of “Chest Pain, *ICD-9*: 786.5” was considered complete concordance; yet no definitive cause of the pain was identified (despite numerous causes likely being ruled out).

There was no relationship between patient age (younger adult versus geriatric), gender, or education level and the category of search term used. Patients who reported talking to their primary care provider prior to presentation did not have a different distribution of search terms than those who did not talk to (or did not identify) a primary care provider (data not shown).

## DISCUSSION

We characterized ED patients’ pre-visit Internet search terms using a qualitative approach and looked at the relationship between these search terms and the patients’ final diagnoses. We found the majority of patients searched online for symptoms rather than for specific diagnoses. Previous studies using web-analytics of Internet queries similarly noted that the majority of searches focused on symptoms.^11^ However, in contrast to studies in other settings, in this sample of ED patients very few searches focused on treatment. For example, in a sample of orthopedic patients 21% sought information about treatment, and in a sample of melanoma patients 96% sought information about treatment.^12^,^13^ The higher ratio of treatment-related searches in the outpatient clinic and specialty context contrasts with our ED data. However, our report is unique in illuminating the frequency and nature of Internet search strategies that may serve as the genesis of the decision to seek unscheduled ED care. Additionally, understanding these symptom-based searches may help EPs address concerns that arise after Internet searches.

Previous data from the Pew Internet and American Life Project highlighted that 41% of “online diagnosers” say that a medical professional confirmed their suspicions, whereas 18% said a medical professional did not agree or offered a different opinion.^1^ We did not specifically query patients about their leading diagnosis post-search; however, in our sample only 11.8% of patients originally searched a diagnosis that perfectly matched their final diagnosis. In contrast, a much higher rate of patients displayed complete (32.3%) or near (30%) concordance between their chief complaint and their discharge diagnosis. This finding may be because the chief complaint represented the patient’s post-search leading diagnosis; however, this metric was likely also influenced by nurse entry of the chief complaint, and the frequent use of symptom-based discharge diagnoses.

The correlation between Google-searched diagnosis and ED diagnosis was poor. One explanation is patients accessing misinformation on the Internet; however, patients’ limited medical knowledge and hypersensitivity to dangerous or deadly diagnoses (e.g., heart attack, stroke) may drive this poor correlation. We are currently in an era of medicine with unprecedented attention to patient satisfaction, and as such, matching patient expectations with experience is necessary for the EP to ensure a satisfied patient. If EPs ask patients about Internet searches and concerns that arose from those searches, the physicians can directly address patients’ concerns and highlight how those concerns have been ruled out. This process may help to match patient experience to their expectations and may ultimately improve patient satisfaction. Future studies measuring the impact of EPs asking about Internet searches, directly addressing patients’ concerns after searching the Internet, and the impact on patient satisfaction are warranted.

Many of the patients in our sample departed the ED with a similar level of specificity in terms of diagnosis as when they presented. EPs see value in ED encounters that do not result in a formal diagnosis. Such visits serve many functions, such as excluding life-threating causes of the symptoms, providing reassurance to patients regarding the severity of illness, and the urgency with which to seek future care. However, for patients seeking a diagnosis these visits (and the associated physicians) may be viewed negatively because the patient’s ultimate question (what is wrong with me?) was not definitively answered. Armed with the knowledge garnered from this study, EPs can better explain to patients the value of an ED visit by asking about Internet searches and addressing the concerning diagnoses patients encountered after searching for their symptoms online.

The lack of a “formal” diagnosis is a frequent occurrence in the ED; a recent study reported that at least 37% of discharged ED patients do not receive a pathologic diagnosis.^14^ Faced with this uncertainty, patients often experience fear and anxiety that negatively influences their mental and physical health in the post-discharge period.^15^,^16^ We did not follow patients after their visit to learn about post-visit Internet searches. However, it seems likely that the lack of a formal diagnosis mentioned above could also be associated with post-visit Internet searches. Bell et al. evaluated the factors patients named as prompting a post-visit Internet search (not specific to ED patients) and found that patients were more likely to use the Internet post-visit when their anxiety was high and their trust in the physician was low.^17^

Interestingly, irrespective of their search terms and concordance, patients used a variety of websites to gather medical information. Although the survey specifically asked patients which destination sites they used to gather information, many volunteered that they used Google to start their search. A similar pattern exists in other settings as well with an estimated eight out of ten health-related Internet searches starting at a search engine such as Google, Yahoo! or Bing.^1^ Interestingly, evaluation of the content of the “top hits” on Google, Yahoo! and Bing searches with respect to critical symptoms that would prompt an acute evaluation revealed that a minority of sites contained a clear set of critical symptoms or recommendations for further care.^18^ These metrics make it easy to criticize such websites for not clearly defining symptoms that warrant emergent evaluation. At the same time, it is difficult to imagine how an online list of symptoms could appropriately capture the nuanced combination of patient risk factors, presenting symptoms, and physical examination (as well as years of clinical experience) that allow physicians to accurately diagnose and risk-stratify patients.

## LIMITATIONS

This was a small sample of patients from a single site, containing English-speaking participants with generally high income, education and ease of access to the Internet. Data collection occurred over an eight-week timeframe on a convenience sample basis during daytime hours and limited to patients who were not too severely ill to participate. Although the decision to omit individuals unable to read the survey may have introduced sampling bias, we believe this to be minimal as we were asking patients about an activity that requires basic writing and reading skills (namely, typing a search term into the Internet and reading the results of the search). These factors limit the generalizability.

We only present data on patients who answered “yes” to performing an Internet search prior to arrival. We did not investigate why patients did not perform an Internet search if they answered “no” and therefore cannot comment on whether this lack of search was related to their clinical condition, Internet access or trust in the Internet. Additionally, there are limitations inherent in the metrics. The patient search terms were based on self-report and are subject to recall bias. In some cases, a prior healthcare encounter (either an established diagnosis or another same-day encounter via phone or in-person) likely influenced the search terms and chief complaint. For example, one patient searched online for “kidney infection” and their chief complaint was “kidney infection, seen yesterday.” Nurse interpretation, as noted above, also potentially influenced the recording of the chief complaints. Although this limitation prevented us from conducting an analysis of the relationship between search terms and chief complaint, it is an accurate reflection of the working environment of the ED.

Finally, the use of *ICD-9* codes is also potentially flawed. In our system the ED attending or resident physician enters the diagnosis into the EMR at either the time of ED note completion or the time of disposition. Variable amounts of information may be available depending on the timing and could result in a less specific *ICD-9* (e.g., viral syndrome instead of influenza). Since the time of data collection, ICD coding has advanced to the currently used *ICD-10* coding system that contains 155,000 codes and procedures compared to only 17,000 codes in the *ICD-9* system.^19^ The expansion of the coding system may have resulted in more specificity in ED discharge diagnoses; however, this topic requires further study.

## CONCLUSION

A quarter of our sample reported using the Internet prior to their ED visit and approximately half used a symptom-based approach for their search strategy. Similarly, nearly half of these patients left the ED with a symptom-based (or non-pathologic) diagnosis. When patients did search for a specific diagnosis, only 29% searched for the diagnosis they eventually received. Physicians who discharge patients with a symptom-based diagnosis may benefit from understanding that patients had similar symptom-based searches prior to coming to the ED, and more fully explain how the ED workup has ruled out specific diagnoses patients were concerned about after an Internet search and changed the treatment plan prior to discharge. Such conversations may address the fear and anxiety that other studies have reported being associated with diagnostic uncertainty at the time of discharge.

## Figures and Tables

**Figure:**
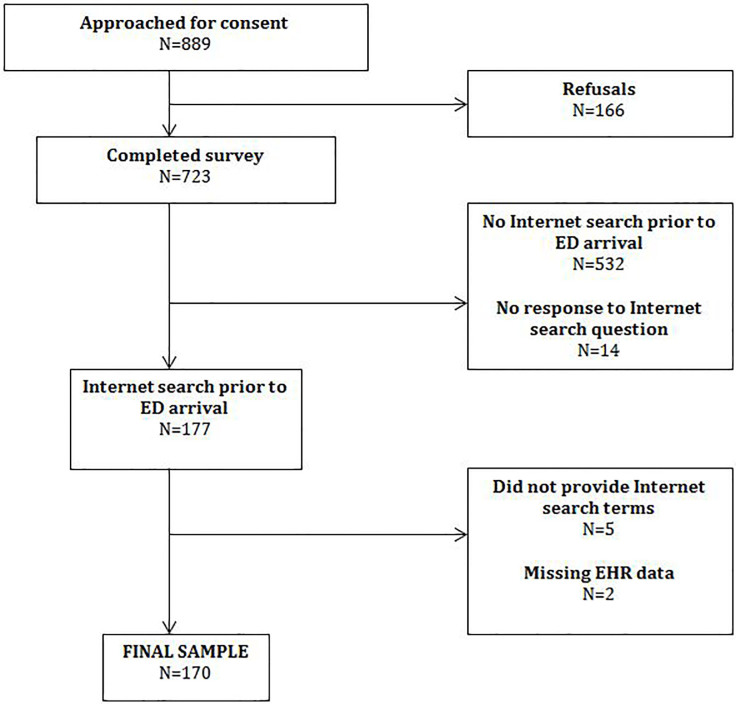
Flowchart of patient enrollment.

**Table 1:** Sample demographics in an analysis of the use of health-related Internet searches by patients prior to presentation at the emergency department (ED).

Variable	n (%)
Age, mean (standard deviation)	47 (18.2)
Geriatric (age >65)	45 (26.5)
Female	99 (58.6)
Race	
African American	33 (19.5)
White	111 (65.7)
Other	25 (14.8)
Education	
High school or less	23 (13.6)
Some college	34 (20.1)
College graduate	59 (34.9)
Advanced degree	53 (31.4)
Household income level ($)	
<50,000	50 (32.9)
50,000–100,000	50 (32.9)
>100,000	52 (34.2)
Triage acuity (ESI)	
2-Emergent	72 (42.3%)
3-Urgent	77 (45.3%)
4-Semi-urgent	21 (12.4%)
ED disposition	
Discharged home	105 (61.8%)
Admit-observation status	32 (18.8%)
Admit-inpatient status	33 (19.4%)
Number of devices owned with internet access	
0	3 (1.8)
1	30 (16.6)
2	52 (30.6)
3	85 (50.0)
Report daily internet use	162(95.9)

*ESI,* Emergency Severity Index.

**Table 2:** Distribution of Internet search terms.

Search term groupings*	Description	n (%)	
Symptom	A search term querying a descriptive symptom, but not a specific diagnosis by name	133 (54.7%)	“Blood in urine” (pt 386) “stiff neck” (pt 768) “pressure in the ears” (pt 610)
Diagnosis	A search term querying a diagnosis by name (and or symptoms related to a diagnosis by name)	77 (31.7%)	“UTI” (pt 452) “meningitis” (pt 459) “heart attack” (pt 375)
Treatment options	A search term querying treatment options for different diagnoses or symptoms	3 (1.2%)	“elbow surgery” (pt 466) “natural alternatives to reduce swelling” (pt 315)
Anatomy	A search term querying items related to anatomy without clear reference to symptoms, diagnosis or treatment	10 (4.1%)	“gall bladder” (pt 725) “stomach” (pt 666)
ED processes or physicians	A search term querying things related to the hospital (e.g., availability of specialists), the emergency department and its processes (e.g., wait time) or specific physicians	1 (0.5%)	“hand doctors” (pt 142)
Other	Questions in which the main topic was unclear or did not fit into any of the above categories	19 (7.8%)	“how often should I check my fever” (pt 495) “colonoscopy and kidney stone correlation” (pt 468)

* N=243 search terms from 170 patients.

*pt*, patient, *ED,* emergency department; *UTI*, urinary tract infection.

**Table 3:** The trajectory between initial Internet search term and final emergency department diagnosis.

Trajectory grouping	Examples from our sample	
		
n (%)	Search term	Final diagnosis (ICD-9)
Flat trajectory—symptoms		
53 (34%) searched symptoms	final diagnosis symptom based	
	abdominal pain→	abdominal pain, other unspecified site (789.09)
	side pain, fever, shaking→	abdominal pain, unspecified site (789)
Flat trajectory—diagnosis		
20 (13%) searched diagnosis→	final “formal” diagnosis (original search correct)	
	COPD→	obstructive chronic bronchitis, with exacerbation (491.21)
33 (21%) searched diagnosis→	final “formal” diagnosis (original search incorrect) Severed tendons hand and wrist→	sprain or strain of unspecified site of wrist (842)
General to specific trajectory		
35 (22%) searched symptoms→	final “formal” diagnosis	
	fever→	pneumonia (486)
Specific to general trajectory		
16 (10%) searched diagnosis→	final diagnosis symptom based	
	stress fracture→	pain in soft tissues of limb (729.5)

N=157 (excluded 13 patients who only searched for treatment, anatomy, ED processes or “other”).

**Table 4:** Concordance between chief complaint on ED presentation and final diagnosis on ED discharge.

			Example from our sample	
			
Concordance grouping	Description	n (%)	Chief complaint	Final diagnosis
No concordance	No relationship between chief complaint and final diagnosis in body system or disease	20 (11.8%)	“Chest pain”	→UTI
			“Headache/Dizziness”	→Rhabdomyolysis
Partial concordance	CC and FD are mostly unrelated, but have one aspect of similarity (e.g., region of the body involved)	44 (25.9%)	“Abdominal pain”	→Malignant neoplasm of bladder
			“SOB”	→Acute Anxiety state, unspecified
Near concordance	CC and FD are mostly unrelated, but have one aspect of similarity (e.g., region of the body involved)	51 (30.0%)	“Right lower quadrant pain”	→Appendicitis
			“Finger injury”	→Closed fracture of the middle or proximal phalanx of the hand
Complete concordance	CC is the same as FD (allowing for differences in medical and lay terminology)	55 (32.3%)	“Numbness L side since yesterday”	→Disturbance of skin sensation
			“Infection to R leg”	→Cellulitis and abscess of Leg
			“Pancreatitis”	→Pancreatitis

*CC*, chief complaint; *FD*, final diagnosis; *L*, left; *R*, right; *SOB*, shortness of breath; *UTI*, urinary tract infection.
